# Gut microbiota signatures and modulation in irritable bowel syndrome

**DOI:** 10.20517/mrr.2021.12

**Published:** 2022-03-05

**Authors:** Giovanni Marasco, Cesare Cremon, Maria Raffaella Barbaro, Vincenzo Stanghellini, Giovanni Barbara

**Affiliations:** ^1^Division of Internal Medicine, IRCCS Azienda Ospedaliero-Universitaria di Bologna, Bologna 40138, Italy.; ^2^Department of Medical and Surgical Sciences, Alma Mater Studiorum Università di Bologna, Bologna 40138, Italy.

**Keywords:** Gut microbiota, metabolome, irritable bowel syndrome, diarrhea

## Abstract

Irritable bowel syndrome (IBS) affects approximately one tenth of the general population and is characterized by abdominal pain associated with abnormalities in bowel habits. Visceral hypersensitivity, abnormal intestinal motor function, mucosal immune activation, and increased intestinal permeability concur to its pathophysiology. Psychological factors can influence symptom perception at the central nervous system level. In addition, recent evidence suggests that dysbiosis may be a key pathophysiological factor in patients with IBS. Increasing understanding of the pathophysiological mechanisms translates into new and more effective therapeutic approaches. Indeed, in line with this evidence, IBS therapies nowadays include agents able to modulate gut microbiota function and composition, such as diet, prebiotics, probiotics, and antibiotics. In addition, in the last decade, an increasing interest in fecal microbiota transplantation has been paid. An in-depth understanding of the intestinal microenvironment through accurate faucal microbiota and metabolite analysis may provide valuable insights into the pathophysiology of IBS, finally shaping new tailored IBS therapies.

## INTRODUCTION

Irritable bowel syndrome (IBS) is a functional gastrointestinal disorder (FGID), now termed disorders of gut-brain interaction (DGBI), characterized by symptoms such as discomfort or abdominal pain and change in stool form or frequency which cannot be explained by the presence of structural or tissue abnormalities^[1]^. IBS is one of the most frequent DGBI, affecting about 3%-5% of the Western population and is associated with reduced quality of life and high social costs^[2]^. IBS pathophysiology remains not completely understood, and this results in unsatisfactory management. For all these reasons, IBS represents a challenge not only for the general practitioner but also for the gastroenterologist^[1]^. Nonetheless, increasing understanding of pathophysiological mechanism of IBS has occurred in the last decades, leading to more successful management^[3]^. Specifically, major advances have been achieved in the detection of microbiota changes and microbiota modulation^[3-7]^. Indeed, gut microbiota is able to shape the host innate and adaptive immune system through specific interactions between gut bacteria and mucosal immune cells. This relationship greatly contributes to immunological homeostasis^[8-10]^. Recent data suggest that IBS pathophysiology includes changes in gastrointestinal motility, intestinal secretion, visceral hypersensitivity, mucosal immune activation, and intestinal permeability, all of which can be influenced by the gut microbiota^[3]^. Briefly, microenvironmental factors, including gut microbiota, may overstimulate the mucosal immune system through the altered intestinal epithelial barrier, inducing abnormal signaling to extrinsic afferent and enteric nerves, and this may affect the intestinal physiology and sensory perception with IBS symptom generation^[11]^. Moreover, IBS symptoms vary according to diet, host genetics, and environment, which can in turn modulate the human gut microbiome^[3]^.

Treatments influencing gut microbiota such as prebiotics, probiotics, and non-absorbable antibiotics have been shown to be safe and effective for the treatment of patients with IBS^[12]^. In this review, we briefly summarize the current understanding on dysbiosis and discuss microbiota modulation in patients with IBS.

## CLINICAL FEATURES AND EPIDEMIOLOGY

IBS is defined by symptom-based diagnostic criteria known as the “Rome criteria”, now currently in their fourth iteration (see Table 1). Patients are categorized according to predominant stool pattern by use of the Bristol Stool Form Scale. The categories are IBS with diarrhea (IBS-D), IBS with constipation (IBS-C), IBS with mixed stool pattern (IBS-M), and IBS unclassified (IBS-U) [Table 1]^[1]^. Supportive symptoms include defecation straining, feeling of incomplete bowel movement, urgency, passing mucus, and bloating. IBS patients also often complain of mood problems; other gastrointestinal symptoms such as functional dyspepsia or gastroesophageal reflux disease; extraintestinal symptoms such as fibromyalgia, headache, and back pain; and genitourinary symptoms, for example, in women, the so-called pelvic pain, worsening of symptoms during menstruation, dyspareunia, or other gynecologic symptoms^[13,14]^. These symptoms increase the severity of IBS and may be associated with psychological factors, including psychiatric disorders (e.g., panic, generalized anxiety, mood, and post-traumatic stress disorders), sleep disturbance, and dysfunctional coping^[15]^. It is difficult to obtain a reliable estimation of IBS prevalence since there are no accepted biomarkers for this condition. Moreover, the prevalence also changes among different geographical regions due to variations in symptom interpretation and reporting^[16]^. A recent a cross-sectional survey of 33 nations promoted by the Rome Foundation^[2]^ reported that IBS prevalence rates range between 1.3% and 7.6%, with a pooled prevalence of 4.1%. using the Rome IV criteria. The peak incidence of IBS was observed in the third and fourth decades of life^[17]^, although in certain countries it is more prevalent in younger men aged 16-30 years^[18]^. Even if it is not a life-threatening condition, IBS significantly impacts quality of life and places a considerable burden on both the individual sufferers and society as a whole^[19]^.

**Table 1:** The Rome IV criteria for IBS and its subgroups (adapted from Ref.^[1]^)

**IBS**	Recurrent abdominal pain, on average for at least one day per week in the past three months, associated with two or more of the following: related to defecation, a change in frequency of stool, a change in stool form. Criteria must be fulfilled for the past three months, with symptom onset at least six months before diagnosis
**IBS with constipation**	< 25% of Bristol Stool Form Types 6 or 7 and ≥ 25% of bowel movements of Bristol Stool Form Types 1 or 2
**IBS with diarrhea**	< 25% of Bristol Stool Form Types 1 or 2 and ≥ 25% of bowel movements of Bristol Stool Form Types 6 or 7
**IBS with mixed stool pattern**	≥ 25% of bowel movements of Bristol Stool Form Types 6 or 7 and ≥ 25% of bowel movements of Bristol Stool Form Types 1 or 2
**IBS unclassified**	Patients with IBS criteria outside subgroups according to Bristol Stool Form type

IBS: Irritable bowel syndrome.

## RISK FACTORS AND NATURAL HISTORY

IBS prevalence is higher in women than men and in subjects aged less than 50 years old according to recent systematic reviews^[17,20]^. This condition is also common in patients with functional somatic disorders such as fibromyalgia and chronic fatigue. An ascertained risk factor for IBS is the history of a previous acute bout of enteric infection^[21,22]^, bacterial, viral, or protozoal, which is now termed post-infection IBS^[6]^. A recent metanalysis^[23]^ reported a four-fold risk increase in developing IBS in individuals exposed to an enteric infection, finding that female sex, severe infection course, antibiotic intake, and previous psychological comorbidities were associated to the development of this condition. Another follow-up study over a 16-year period reported an IBS prevalence of 36.8% in a cohort with culture-proven *Salmonella enteritidis* infection^[21]^.

Focusing on disease course, IBS has a fluctuating course over time, mainly in terms of bowel habits^[24]^. IBS patients complain during their lifetime of an impaired quality of life^[25]^ and reduced work productivity and social integration^[26]^. Moreover, IBS is associated with anxiety and depression^[27]^, which may in turn worsen patients quality of life and exacerbate gut-related symptom somatization, influencing the way patients perceive their illness^[28]^.

## DIAGNOSIS

As no specific biomarkers of IBS are currently available, the diagnosis is based on patient’s self-reported symptoms, through the use of validated questionnaires. The most recent guidelines^[12,29]^ recommend a positive IBS diagnosis using the Rome criteria (see Table 1) and Bristol Stool Form Scale, which can also be used to direct treatment^[12,29]^, in absence of warning signs by means of clinical history, physical examination, and a limited number of routine laboratory studies. Recognized warning signs are fever, gastrointestinal bleeding, anemia, weight loss, abdominal mass, fever, family history of colon cancer, inflammatory bowel disease, celiac disease, onset of symptoms after 50 years of age, and a major and recent change in bowel function^[30]^. Colonoscopy should be performed in selected patients over 40 or 50 years old with or without alarm features, respectively^[31]^. Among laboratory tests required, a full blood count to exclude colorectal cancer, C-reactive protein to exclude inflammatory bowel diseases, and serological screening for coeliac disease should be performed^[12,29]^.

## PATHOPHYSIOLOGY

**Figure 1:**
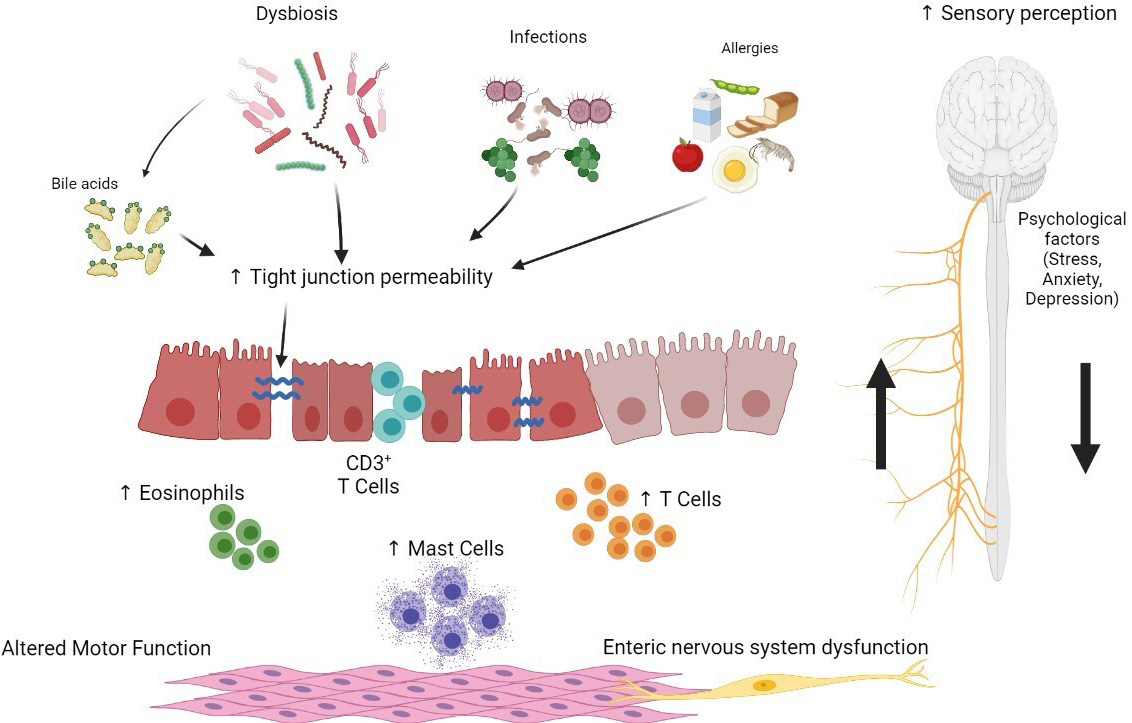
Model of IBS pathogenesis including the complex interplay between peripheral and central factors. IBS: Irritable bowel syndrome.

IBS is a multifactorial disorder with a complex and not completely understood pathophysiology^[31]^. Previously, IBS has been mainly considered a psychosomatic disorder in which psychosocial factors, including stress, anxiety, and depression, markedly contributed to altered motility and visceral hypersensitivity^[32]^. IBS is now considered a disorder of the gut-brain axis (DGBI) characterized by a complex interplay between peripheral and central factors, including genetic predisposition, alterations in gastrointestinal sensory-motor function, intestinal dysbiosis, increased intestinal permeability, mucosal low-grade inflammation and immune activation, neuroendocrine abnormalities, food sensitivity, and psychosocial factors [Figure 1]^[11,31,33]^. In particular, the current view suggests that, in genetically predisposed individuals, triggers such as early-life adverse events, psychological factors, or a previous episode of infectious gastroenteritis induce persistent alterations in the mucosal barrier, intestinal microenvironment, mucosal immune system, and the enteric nervous system, which controls sensory-motor and secretory functions, leading to symptom generation^[11,31]^. To date, infectious gastroenteritis is the strongest known risk factor for IBS^[6]^.

Because many patients with IBS report an association between their symptoms and eating or eliminating particular foods, it can be hypothesized that diet and intestinal microenvironment, in particular gut microbiota, are central in IBS pathophysiology^[11]^. There are at least three clinical scenarios that link IBS with the gut microbiota, all of which are characterized by disruption of the balance between the host and the intestinal microbial ecosystem: (1) more than 10% of patients with IBS develop their symptoms after an acute episode of infectious (e.g., bacterial, viral, or parasitic) gastroenteritis, the so-called post-infection IBS^[6,11]^; (2) disruption of gut microbiota with systemic antibiotics increases the risk of functional abdominal symptoms, including IBS^[34]^; and (3) treatments aimed at modifying gut microbiota composition, including prebiotics, probiotics, poorly absorbable antibiotics, or fecal microbiota transplantation (FMT), can improve symptoms at least in a proportion of patients with IBS (see below)^[35]^. Thus, dysbiosis, occurring when the diversity, composition, and/or functions of the intestinal microbial ecosystem are disrupted, could contribute to the alteration of the usual intestinal functions with implications in the development, progression, and symptom flare-up of several diseases^[11]^. IBS is characterized by a well-established microbiota dysbiosis associated with impairment of the intestinal physiology^[1]^. The interplay between the host and the gut microbiota is constantly challenged by numerous factors, including environment, food, and immunological factors. A recent report of the Rome Foundation on the intestinal microenvironment and DGBI provides an excellent overview of the importance of the environment, including food, diet, bile acids, microbiota, and their metabolic interactions, in the pathophysiology and symptom generation of patients with DGBI in general, and IBS in particular^[11]^.

## GUT MICROBIOTA CHARACTERIZATION

The human gut harbors a community of about 10^14^ microorganisms resulting from thousands of years of co-evolution with the host, with an intricate and mutually beneficial relationship^[36]^. In particular, the human intestinal microbial ecosystem consists of over 1000 different microbial species belonging to five predominant phyla: *Firmicutes* and *Bacteroidetes* followed by *Actinobacteria*, *Verrucomicrobia*, and *Proteobacteria*^[37]^. One of the most frequent slogans in the past years has been that the human body contains 10 times more microorganisms than human cells^[38]^. However, this was a rough calculation performed several years ago, and we now know that the ratio of microbial to human cells is probably closer to 1:1^[39]^. The gut microbiota is an extremely complex community, in which all the domains of life are represented, including not only bacteria, which are the predominant and the most studied to date component, but also archaea, micro-eukaryotes (i.e., fungi and protists), and viruses^[40,41]^. Gut microbiota participates in digestive functions, shapes the host immune system, modulates host metabolisms, and influences local and systemic processes, such as vitamin intake and nutrient metabolism^[42,43]^. The intestinal microbial ecosystem also protects against pathogens, by competing with them for nutrient uptake and regulating host immunity^[44]^.

## STRUCTURE OF THE GUT MICROBIOTA IN IBS

Recently, with the advent of culture-independent next-generation sequencing techniques, several studies have been aimed at characterizing the composition of gut microbiota in patients with DGBI and in particular with IBS. Despite conflicting results, most studies have shown a reduced microbial diversity, particularly in patients with IBS-D, suggesting that the microbial species involved in maintaining homeostasis may be missing^[45,46]^; interestingly, some data suggest an association between lower microbial diversity and looser stool, suggesting a potential relevance in the pathophysiology of diarrhea and even fecal incontinence^[47]^.

In a recent systematic review involving 777 patients with IBS and 461 healthy controls, most of the studies agreed in showing increased *Firmicutes* and decreased *Bacteroidetes*, associated with an increased *Firmicutes*/*Bacteoridetes* ratio; however, at lower taxonomic levels, the data are inconsistent^[48]^. Recently, a systematic review identified potentially harmful and protective bacteria in patients IBS^[49]^. Potentially harmful bacteria belong to the families *Enterobacteriaceae* (phylum *Proteobacteria*) and *Lactobacillaceae* (phylum *Firmicutes*) and genus *Bacteroides* (phylum *Bacteroidetes*). In particular, *Bacteroides* were found to be increased in patients with IBS-D and were associated with mucosal low-grade inflammation. Conversely, protective bacteria belong to *Clostridiales*, especially *Faecalibacterium prausnitzii*, an anaerobic butyrate-producing bacteria which was studied for its favorable anti-inflammatory role and ability to help maintain gut barrier homeostasis^[49]^. Gut microbiota composition has also been related to symptom severity of patients with IBS. In particular, a recent study first linked symptom severity to characteristic alterations of gut microbiota, including reduced diversity, reduced exhaled methane, relative reduction of *Methanobacteriales* and *Prevotella* enterotype, and abundance of *Bacteroides* enterotype^[50]^.

## FUNCTION OF THE GUT MICROBIOTA IN IBS

The lack of a clear association between gut microbiota alterations and IBS and the recent advances in omics-based analyses of microbiota (including metabolomics and metagenomics) have led to the idea that symptoms could be associated with modifications of the function more than the structure of gut microbiota. In fact, a comprehensive longitudinal analyses of IBS patients revealed a role for the gut microbiota in modulating metabolism and influencing host gastrointestinal function, especially for IBS-C patients, who exhibited a more distinct gut microbiota profile and a greater variability of over time compared to healthy and IBS-D subjects^[51]^. Among the multiple products of bacterial metabolism, the importance of the short-chain fatty acids (SCFAs) in colonic homeostasis has been globally acknowledged^[52]^. SCFAs are the product of carbohydrate fermentation by commensal anaerobic bacteria, and they play a role in preserving gut barrier functions and modulating immune function and have anti-inflammatory properties^[52]^. We previously reported reduced levels of SCFAs in IBS-C compared to IBS-U and IBS-D patients, associated with a diverse representation of *Clostridiales* operational taxonomical units^[53]^. Moreover, SCFA levels were in turn associated with the Bristol Stool Scale Form and reduced levels in IBS-C patients positively correlated with several cytokines (particularly IL-10) and were negatively correlated with IgA levels^[53]^. Besides, a recent meta-analysis identified low levels of propionate and butyrate in feces from patients with IBS-C, as compared with healthy controls, and a higher proportion of butyrate in fecal samples of patients with IBS-D, as compared with controls^[54]^. Interestingly, some authors have suggested a role of SCFA-producing bacteria such as *Ruminococcaceae*, unknown *Clostridiales*, and *Erysipelotrichaceae* in the dysbiosis of patients with IBS, particularly with IBS-D^[55]^. In accordance with these data, the reduction of butyrate-producing bacteria would result in decreased availability of butyrate and, consequently, in the loss of its protective action on the intestinal epithelial barrier. Other studies have found *Ruminococcus* to be increased in IBS patients, suggesting even a potential role as a biomarker of this disorder^[56]^. Interestingly, this represents a potential attractive therapeutic target in IBS, as suggested by a recent study showing that *Lactobacillus paracasei* CNCM I-1572 improves symptoms, modulates gut microbiota structure and function through the reduction of *Rumicococcus* with consequent increased levels of acetate and butyrate, and reduces intestinal immune activation in patients with IBS^[57]^.

A recent integrated and longitudinal multi-omic analysis of IBS patients revealed a role for the gut microbiota in modulating purine metabolism and influencing host gastrointestinal function, identifying purine starvation as a possible therapeutic target in IBS^[51]^. Besides, analyzing IBS subtype-specific and symptom-related variations in microbial composition and function, the authors found a reduction in SCFAs associated with IBS-C and an increase in stool tryptamine, capable of increasing fluid secretion and decreasing transit time, and of unconjugated primary bile acids in stool samples of patients with IBS-D^[51]^. Another recent study used a commercially available kit for gut microbiome analysis, finding a clear separation between IBS patients and healthy subjects through distinctive metabolites patterns. Furthermore, patients with IBS had a distinct fecal microbiota and metabolite profile linked to bowel habits^[58]^.

To conclude, it is necessary to specify that unfortunately, to date, results of the abovementioned studies often are inconsistent and sometimes contradictory. Even though no clear microbiota signature nor metabolomic or metagenomic alterations can be related to IBS yet, all together these data suggest a role of dysbiosis in IBS pathophysiology and symptom generation, although more work needs to be done to better clarify all these aspects. Furthermore, these alterations constitute the rationale and starting point for pharmacological and non-pharmacological treatments aimed at modulating the intestinal microbiota in patients with IBS.

## GUT MICROBIOTA MODULATION

Increasing evidence suggests a key role of microbial modification approaches, including diet, pre- and probiotics, poorly absorbable antibiotics, or FMT, in the management of patients with IBS^[59]^.

## DIET

Since gut microbiota is deeply influenced by diet, a dietary approach represents an interesting first-line treatment opportunity for IBS with a potential impact on gut microbiome.

Several randomized controlled trials and some meta-analyses^[60,61]^ have demonstrated that a low fermentable oligo-, di-, and mono-saccharides and polyols (FODMAP) diet reduces fermentation, improving global symptoms as well as abdominal pain in patients with IBS, particularly with IBS-D, when compared with habitual diet or different dietary interventions^[60,61]^.

A recent study showed that about 50% of IBS cases manifested a “pathogenic” gut microbial signature that showed an enhanced clinical responsiveness to the dietary therapy shifting towards the healthy profile on the low FODMAP diet, with an increase in Bacteroidetes (*P* = 0.009), a decrease in Firmicutes species (*P* = 0.004), and normalization of primary metabolic genes^[62]^.

However, due to the impact of this alimentary approach on the intestinal microbiota with potential deleterious alterations in the microbiome, FODMAPs should be reintroduced to tolerance after a limited period of restriction^[63]^. In addition, long-term studies are still inconclusive, suggesting that this dietary approach warrants future studies. A low FODMAP diet should be recommended as a second-line diet for IBS, after a traditional dietary advice to eat small regular meals, avoid known trigger foods, and reduce alcohol and caffeine^[29]^. In addition, novel trends in dietary advices should be investigated in terms of gut microbiota modifications, such as the recent evidence regarding the effectiveness of green kiwifruit for treating IBS-C, as also assessed by the European Food Safety Authority^[64,65]^.

## PRE- AND PRO-BIOTICS

A recent systematic review with meta-analysis assessed the role of prebiotics, probiotics, and synbiotics in the management of patients with IBS^[35]^. Prebiotics are a substrate that is selectively utilized by host microorganisms conferring a health benefit^[66]^. They are non-digestible food ingredients, mostly carbohydrates, that stimulate the growth and/or activity of health-promoting bacteria. Synbiotics refer to dietary supplements combining probiotics and prebiotics in a form of synergism. Due to the paucity of data and the low-quality of trials assessing these approaches, a definitive conclusion on the efficacy of prebiotics or synbiotics in IBS cannot be made at this time^[35]^.

Probiotics are live microorganisms that, when administered in adequate amounts, confer a health benefit on the host^[67]^. Major claims of probiotics in DGBI include modulation of gastrointestinal motility, reduction of visceral hypersensitivity and pain as well as low grade mucosal immune activation, improvement of epithelial permeability, enhancement of gut-brain communication, and modulation of structure and function of gut microbiota with potential impact on restoring intestinal dysbiosis, all mechanisms involved in IBS pathophysiology^[67-69]^. However, most of these claims are based on convincing experimental evidence from preclinical or pilot studies, and their clinical efficacy is still matter of debate^[68]^. The inconsistent results are also likely due to our limited knowledge of the genetic and mechanistic bases of the interaction between microbial taxa and the host. Future research should focus on better understanding strain-specific features of the different probiotics. A recent technical review on the role of probiotics in the management of gastrointestinal disorders by the American Gastroenterology Association found that there was either insufficient evidence to recommend the use of probiotics as a part of clinical practice or a significant knowledge gap precluding any conclusions^[70,71]^. Although the most recent international guidelines suggest against the use of probiotics for the treatment of global IBS symptoms^[12,70,71]^ or only in the context of a clinical trial, recent well-performed trials using well-defined end-points as recommended by the European Medicines Agency (EMA) show promising results in patients with IBS^[57,72]^. Based on these data, the most recent British Society of Gastroenterology guidelines on the management of IBS suggest that probiotics may be an effective treatment for global symptoms and abdominal pain in IBS, although a specific species or strain could not be recommend^[29]^. Further studies are needed to clarify definitively the potential role of probiotics in the management of patients with IBS.

## EUBIOTICS

Rifaximin, a poorly absorbable non-systemic antibiotic derived from rifamycin, is the best studied and only the United State Food and Drug Administration (FDA)-approved antibiotic for the treatment of patients with IBS-D. In two identically designed, phase 3, placebo-controlled studies (TARGET 1 and 2, involving 1260 subjects), rifaximin, at a dose of 550 mg three times daily for two weeks, was more effective than placebo in significantly reducing IBS global symptoms, bloating, abdominal pain, and loose or watery stools of patients with IBS without constipation^[73]^. A third phase 3 trial (TARGET 3) involving 636 patients with IBS-D according to Rome III criteria clarified definitively that repeat treatment with rifaximin is effective and safe, without developing on-going bacterial resistance or significant alterations in the microbiome^[74]^. The main international guidelines on IBS suggest the use of rifaximin to treat global symptoms of patients with IBS-D^[12,29]^. Rifaximin can reduce bacterial virulence and translocation, as well as exerting an anti-inflammatory effect. It is also able to modulate the gut microbiota, favoring the growth of “healthy-promoter” bacteria without altering its overall composition^[75,76]^. Several metagenomic analyses reported an increase in Bifidobacterium, Faecalibacterium prausnitzii, and Lactobacillus abundance after rifaximin treatment. Therefore, rifaximin can be defined as a “eubiotic”, i.e., a positive modulator of the gut ecosystem^[75,76]^.

## FMT

FMT, the process of transferring intestinal microbiota from a healthy donor into the gastrointestinal tract of a patient with dysbiosis, a proven effective treatment of recurrent *Clostridioides difficile *infection^[77]^, has been suggested and evaluated in IBS^[78]^. Unfortunately, in IBS, the data are mixed, probably due to the variety of routes of administration, formulations, and number and type of donors^[79]^. Recently, a large, single-center trial including IBS of all subtypes showed that FMT (30 and 60 g), acquired from a single so-called super-donor who was in excellent health, and delivered into the distal duodenum, was significantly more effective than placebo (autologous FMT)^[80]^. In particular, after three months, the responder rate was 76% in the 30 g FMT group and 89% in the 60 g FMT group, as compared with 24% in the autologous FMT group, with similar differences using both the FDA and the EMA responder endpoints. Further large trials are needed to clarify whether FMT is effective and safe in the management of patients with IBS.

## CONCLUSION

IBS is a frequent clinical condition characterized by pain and bowel habit abnormalities. Our understanding of IBS pathophysiology has enormously improved over the last two decades. IBS patients show imbalances in gut microbiota composition and function, which may elicit symptoms development. Additionally, patients with IBS benefit from gut microbiota modulating therapies. Longitudinal studies showing gut microbiota fluctuation over time and dietary habits have helped understand IBS microbial signatures, identifying metabolic pathway that may be responsible of symptoms development that can be targeted in future. However, further longitudinal integrated multi-omics studies and functional investigation of the genetic bases and mechanisms involved in gut microbiota-host interplay are needed to extend our understanding of the role of gut colonizers in IBS to elaborate more targeted dietary and therapeutic approaches for this disease and, finally, develop microbiota-targeted therapies.

## DECLARATIONS

### Copyright

© The Author(s) 2022
